# Investigating intermolecular interactions among CO_2_, water and PEEK-ionene membrane using cryo ToF-SIMS and isotopic labeling

**DOI:** 10.3389/fchem.2025.1564084

**Published:** 2025-03-14

**Authors:** Jennifer Yao, Jeffrey A. Dhas, Lyndi E. Strange, Jason E. Bara, Sudhir Ravula, Eric D. Walter, Ying Chen, David J. Heldebrant, Zihua Zhu

**Affiliations:** ^1^ Pacific Northwest National Laboratory, Richland, WA, United States; ^2^ University of Alabama, Department of Chemical & Biological Engineering, Tuscaloosa, AL, United States; ^3^ Washington State University, Department of Chemical Engineering and Bioengineering, Pullman, WA, United States

**Keywords:** cryo ToF-SIMS, PEEK-ionene membrane, intermolecular interactions, isotopic labelling, gas separation, ionic liquid

## Abstract

Cryogenic time-of-flight secondary ion mass spectrometry (cryo ToF-SIMS) has emerged as a powerful tool for investigating molecular interactions, speciation, and dynamics in materials for CO_2_ capture. In this study, we apply cryo ToF-SIMS to probe interactions between CO_2_, water, and PEEK-ionene membranes—a promising material for direct CO_2_ capture due to its selectivity, durability, and efficiency. Despite this potential, the mechanisms governing CO_2_ diffusion and the influence of water vapor on CO_2_ behavior remain unclear. To address this, we loaded PEEK-ionene membranes with ^13^CO_2_ and D_2_O and employed cryo ToF-SIMS to visualize the 3D distribution of CO_2_ and water within the membrane. While prior studies suggest that ^13^CO_2_ is absorbed under ambient conditions, our cryo ToF-SIMS analysis revealed no enhancement of the ^13^C/^12^C ratio, suggesting weak CO_2_-membrane interactions. As a result, CO_2_ vaporizes even at low temperatures (−140°C) under vacuum conditions. In contrast, D_2_O displayed a relatively homogeneous distribution in the membrane, suggesting stronger water-membrane interactions via hydrogen bonding (18–20 kJ/mol). Interestingly, CO_2_ was not detected in D_2_O-loaded membranes, indicating minimal interference from water vapor on CO_2_ diffusion. As a comparison, the cryo ToF-SIMS data show that ^13^CO_2_ can readily react with a basic Na_2_CO_3_ aqueous solution to form NaH^13^CO_3_. These findings demonstrate cryo ToF-SIMS as a critical technique for understanding gas-water-membrane interactions, offering insights for membrane functionalization to improve CO_2_ capture efficiency.

## 1 Introduction

Climate change and global warming have led to an urgent need for technologies capable of capturing and storing CO_2_ from industrial processes and the atmosphere. Among the various approaches developed to address this challenge, membrane-based separation has emerged as a promising solution due to its potential for high selectivity, efficiency, and adaptability to various operational environments (Kammakakam et al., 2022; Jansen and Drioli, 2009).

Poly (ether ether ketone) (PEEK) is a semicrystalline thermoplastic known for its exceptional thermal stability, mechanical properties, and chemical resistance, making it a prime candidate for demanding applications. While pristine PEEK exhibits low gas permeability and is not considered a viable polymer for gas separation membranes, development of PEEK-ionene membranes offers opportunities to increase permeability while maintaining a robust polymer structure. PEEK-ionene materials integrate cationic groups directly within their backbones, combining the robustness of PEEK with the selective permeability offered by ionenes. The incorporation of ionic liquids (ILs) further enhances the membranes’ CO_2_ selectivity, making them particularly interesting for gas separation applications (Kammakakam et al., 2022; O’Harra et al., 2022).

Understanding the interactions among CO_2_, PEEK-ionene membranes, and water is essential for improving membrane-based CO_2_ capture technologies. However, it has long been challenging to track small molecules, such as CO_2_ and H_2_O, in organic matrixes at micron or submicron scales. For example, Nuclear Magnetic Resonance (NMR) is a powerful tool which has been extensively used in CO_2_ capture studies for quite a few years (Heldebrant et al., 2008; Heldebrant et al., 2017; Leclaire et al., 2024). It can not only quantitatively monitor CO_2_ in bulk samples, but also identify reactants and products to elucidate reaction mechanisms during the CO_2_ capture process based on chemical shift (Leclaire et al., 2024). However, the spatial resolution of NMR imaging is a limitation, and NMR 3D imaging at micron to submicron scales has been less reported. Transmission electron microscopy (TEM) and scanning electron microscope (SEM) are popularly used imaging tools, and they can provide atomic-level spatial resolution (Winey et al., 2014; Franken et al., 2020; Kourkoutis et al., 2012). However, electron beam damage of organic species and lack of molecular recognition capability make them hardly used to image small molecules in an organic matrix.

To address these challenges, cryo-secondary ion mass spectrometry (cryo-SIMS) is emerging as an effective tool to study the speciation, movement, and exchange of small molecules like CO_2_ and H_2_O within materials. A combination of isotopic labeling and cryo-SIMS provides a unique method to track small molecules in organic matrixes at nanoscale (Hoppe et al., 2013; Gao et al., 2016). SIMS provides 3D nanoscale imaging of elemental, isotopic and molecular species with excellent sensitivity (Williams, 1985; Benninghoven, 1994; Lombardo et al., 2023). The depth resolution of SIMS depth profiling can go down to a few nanometer (nm) in organic matrices and such a capability allows quantitative determination of polymer self-diffusion with a combination of isotopic labeling (Zheng et al., 1997; Zheng et al., 1995). At the same time, SIMS can provide sub-micron lateral resolution isotopic imaging, and such a capability has been used to resolve important scientific questions (Dekas et al., 2009; Pareek et al., 2020; Pett-Ridge and Weber, 2022; Li et al., 2023). More interestingly, ToF (time-of-flight)-SIMS can detect molecular ion and characteristic fragment ions, providing direct molecular evidence to elucidate reaction mechanisms. For example, *in situ* 3D ToF-SIMS imaging of isotopic labeled amino acids in single cells can directly visualize the *de novo* purine biosynthesis process, confirming a long-existing hypothesis that metabolons should be biosynthetic “hotspots” (Pareek et al., 2020).

SIMS operates under high vacuum conditions, making sample preparation crucial, particularly for samples with volatile components (Benninghoven, 1994; Gao et al., 2016). For example, most fresh biological samples contain a high-water content, which poses a challenge for SIMS analysis under ambient conditions. The high vapor pressure of water can interfere with the vacuum environment required for reliable SIMS measurements, leading to signal instability and reduced sensitivity. It is well-known that the vapor pressure is temperature-dependent, and low temperature can greatly reduce vapor pressure. Therefore, cryo-sample preparation has been extensively used in SIMS imaging of biological samples. Studies have shown that cooling biological samples to −110°C can reduce the vapor pressure of water to the 10^–8^ mbar range, enabling standard SIMS analysis (Guo et al., 2020; Cannon et al., 2000).

It should be noted that vapor pressure is closely related to intermolecular interactions. Strong interactions, such metallic bonds and ionic bonds (in metal and metal oxide samples), lead to very low vapor pressure, and such samples can be directly analyzed using SIMS. However, weak intermolecular interactions, such as London dispersion forces, facilitate easier vaporization resulting in high vapor pressure, especially for small gas molecules (e.g., CO_2_, O_2_, N_2_, etc.). Although water molecules are also small, they are unique due to the presence of hydrogen bonds, which have an energy of approximately 18–20 kJ/mol. As a result, water exhibits a relatively low vapor pressure compared to CO_2_ and O_2_. Therefore, temperature during SIMS analysis can be used as an indicator of the strength of intermolecular interactions. For example, water can be retained on the sample surface at −110°C during SIMS analysis. If CO_2_ cannot be retained under the same conditions, it suggests that the CO_2_-sample (polymer + IL) interaction is weaker than hydrogen bonds in water.

In this work, ToF-SIMS 3D imaging was used to investigate PEEK-ionene membranes purged with ^13^CO_2_ and heavy water deuterium oxide (D_2_O). By using isotopically labeled ^13^CO_2_ and D_2_O, we track the interactions of ^13^CO_2_ and D_2_O within the membrane, offering insights into gas sorption and diffusion mechanisms. Additionally, by exploring the effect of water on CO_2_ capture, the study seeks to address potential challenges posed by water vapor in gas separation processes.

## 2 Materials and methods

### 2.1 Materials and preparation

The lecture bottle containing ^13^CO_2_ (99 atom% ^13^C) was purchased from Cambridge Isotope Laboratories, Inc. Sodium bicarbonate (NaHCO_3_) was purchased from Sigma-Aldrich (Catalog Number S5761). Sodium carbonate (Na_2_CO_3_) was obtained from Sigma-Aldrich (Catalog Number 451614). Deuterium oxide (D_2_O) was acquired from Sigma-Aldrich (Catalog Number 151882, 99.9 atom% D).

**FIGURE 1:**
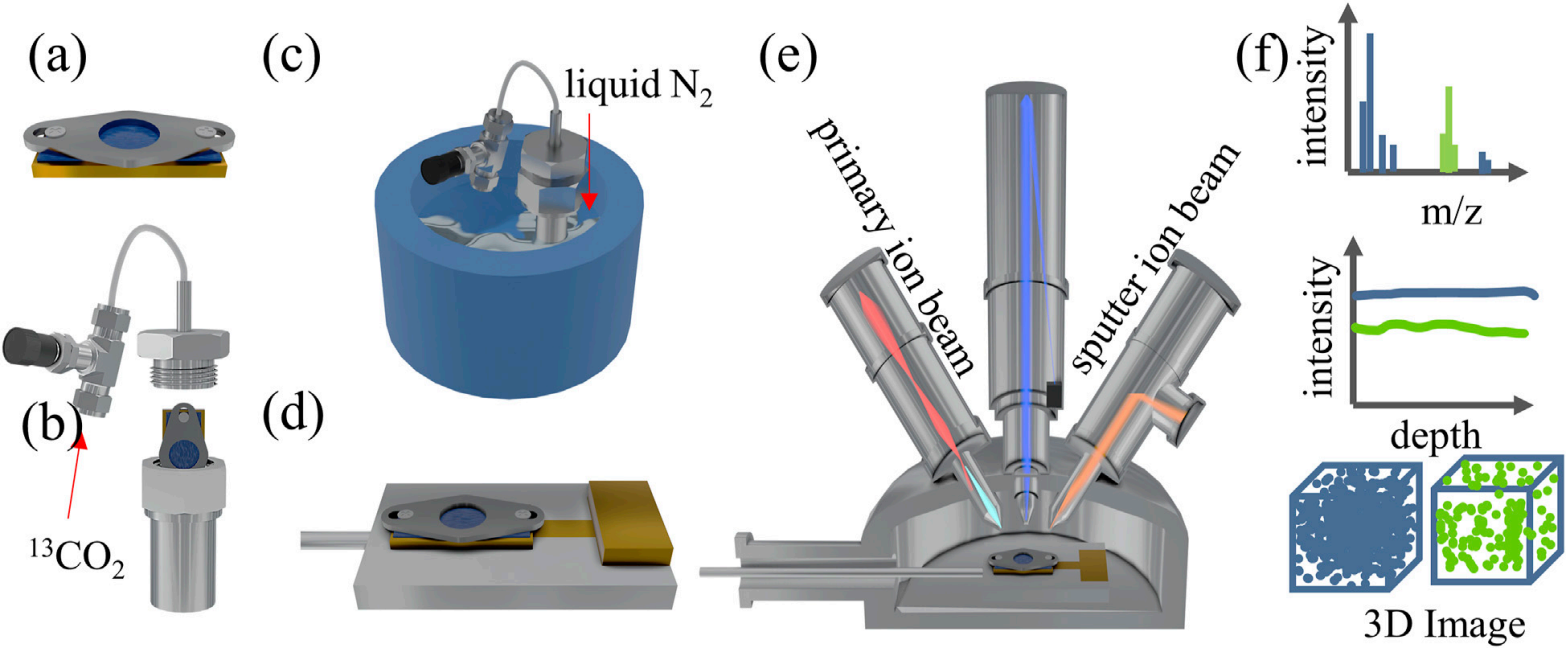
Schematic of cryo ToF-SIMS workflow including the **(a)** sample mounting, **(b)** gas loading, **(c)** flash freezing, **(d)** mounting the sample on a pre-cooled heating/cooling sample holder, **(e)** cryo ToF-SIMS analysis, and **(f)** the results of SIMS spectra, depth profiles, 3D images.

In this study, it is crucial to cool the PEEK-ionene sample after loading ^13^CO_2_. To achieve this, a specially designed sample block was developed to facilitate the pre-cooling before SIMS analysis (Figure 1a). The sample block has an effective area of 8 mm × 16 mm. A 0.1 mm thick molybdenum (Mo) plate, designed with a central hole of 5.0 mm in diameter for sample exposure, is positioned on top of the block. The plate is securely fastened to the copper (Cu) block using two small screw holes, pressing the sample firmly onto the Cu block. This ensures effective thermal contact, allowing efficient cooling to the sample when the sample block is mounted on the heating/cooling sample stage of the SIMS instrument (Supplementary Figure S1). The PEEK-ionene sample was cut into 0.5 cm × 1 cm piece and clamped onto the sample block below the Mo plate (Figure 1a). The sample block was then inserted into a gas loading unit and sealed before purging with 300 psig ^13^CO_2_ (Figure 1b). After purging, the entire gas loading unit was immersed in a liquid nitrogen (N_2_)-filled cryogenic container (Figure 1c) for fast freezing. Once the boiling and bubbling of liquid N_2_ subsided, the membraned-loaded sample block was taken out from the gas loading unit inside the liquid N_2_, covered with a small Cu piece (to avoid any ice condensation during transfer, as shown in Supplementary Figure S1), and then quickly transferred to the pre-cooled cooling/heating sample stage (−140 to −150°C) inside the ToF-SIMS loadlock (Figure 1d). The loadlock pumping process can be started immediately. After the loadlock was pumped down to 1 × 10^−6^ mbar and maintained at the desired temperature (−140 to −150°C), the cooling/heating stage was transferred to the pre-cooled main analysis chamber for ToF-SIMS analysis (Figures 1e, f). The sample temperature during ToF-SIMS analysis was approximately −140°C. It is important to note that the anti-ice condensation Cu piece must be removed by rotating the heating/cooling sample stage, thereby exposing the sample surface before transferring the sample stage into the analysis chamber.

0.1M Na_2_CO_3_ and 0.1M NaHCO_3_ were prepared separately using Milli-Q deionized water (18.2 MΩ.cm at 25°C). 2 mL Na_2_CO_3_ was added into a gas loading apparatus and purged with 300 psig ^13^CO_2_. The solution was left to equilibrate for 2 days. 20 μL of ^13^CO_2_ – reacted Na_2_CO_3_ was taken from the gas loading apparatus and dispensed onto a cleaned silicon (Si) chip which was mounted on the customized sample holder (Figure 1a). The silicon chip was quickly placed into the liquid N_2_-filled cryogenic container for flash freezing. Additionally, 20 µL of 0.1M NaHCO_3_ and 20 µL of 0.1M Na_2_CO_3_ were separately deposited onto two different Si chips directly before flashing freezing. The remaining steps of the procedure follow those illustrated in Figures 1d–f.

The imidazolium-functionalized PEEK–ionene with bistriflimide ([Tf_2_N]) anions were synthesized and the method was reported previously (Ravula et al., 2024; O’Harra et al., 2022). In this study, the membrane *p* ([K (EEK)_2_ (2mIm)_4_(C_6_)_3_][Tf_2_N]_4_ was added with stoichiometric equivalent amount of “free” [C_4_mIm][Tf_2_N], i.e., 1-butyl-3-methylimidazolium bistriflimide ionic liquid (IL).

### 2.2 Cryo ToF-SIMS analysis

In this study, isotope imaging and depth profiling were conducted using a ToF-SIMS 5 mass spectrometer manufactured by IONTOF GmbH, Münster, Germany. To eliminate any thin ice layers or other potential surface contaminants, an Ar cluster ion beam (10 keV, 4.0 nA) was employed to pre-sputter a selected sample surface area of 1,000 × 1,000 μm^2^ for 20 s.

The depth profiling measurements were carried out in the negative ion mode for most of the samples, and both negative and positive ion mode for selected samples. The charge compensation was done with a low energy electron flood gun. Depth profiling of the samples was performed in the non-interlaced mode (analysis 3.2 s, sputter 5.0 s, pulse 1.0 s). For PEEK-ionene membrane samples, a 2.0 keV Cs^+^ beam was used as the sputtering beam, which was scanned on a 500 μm^2^ × 500 μm^2^ area. Cs^+^ sputter beam is chosen because it enhances the negative ionization (Houssiau and Mine, 2010), and ions like C^−^ and H^−^ are of significant interest in this study. The instrument was equipped with a 25 keV Bi_n_^q+^ source (n = 1, 3, and 5, q = 1 and 2). In this study, we used Bi_3_^2+^ beam, which results in a total acceleration voltage of 50 keV. The Bi_3_^2+^ beam was scanned on a 100 μm^2^ × 100 μm^2^ area at the center of the Cs^+^ sputter area. The Bi_3_^2+^ beam scanning was performed in random mode with 100 µs cycle time, 128 × 128 pixels, one shot/frame/pixel and two frames per step. The obtained mass resolution m/Δm was above 7,000 at peak m/z C_2_H^−^ for all measurements. Internal mass calibration was performed using H^−^, C^−^, and C_2_^−^ mass signals in negative ion mode.

After depth profiling, the sputter crater was measured by a Veeco Dektak 6M stylus profilometer. The obtained depth was used to determine the erosion rate (nm/s), which was applied to all measurements of the membrane samples for calibrating the depth profiles, assuming a constant erosion rate. Data analysis of the ToF-SIMS measurements was done using Surface Lab Software version 7.1 (IONTOF GmbH, Münster, Germany). For the Na_2_CO_3_, NaHCO_3_ and ^13^CO_2_-loaded Na_2_CO_3_ aqueous solution samples, the Ar cluster beam was used as a sputter ion beam, because molecular signals were desired.

In addition, a Keyence 3D Surface Profiler (VK-X3000) was applied to obtain the morphological variation of IL-loaded PEEK-ionene membrane before and after ^13^CO_2_ loading.

## 3 Results and discussion

**FIGURE 2:**
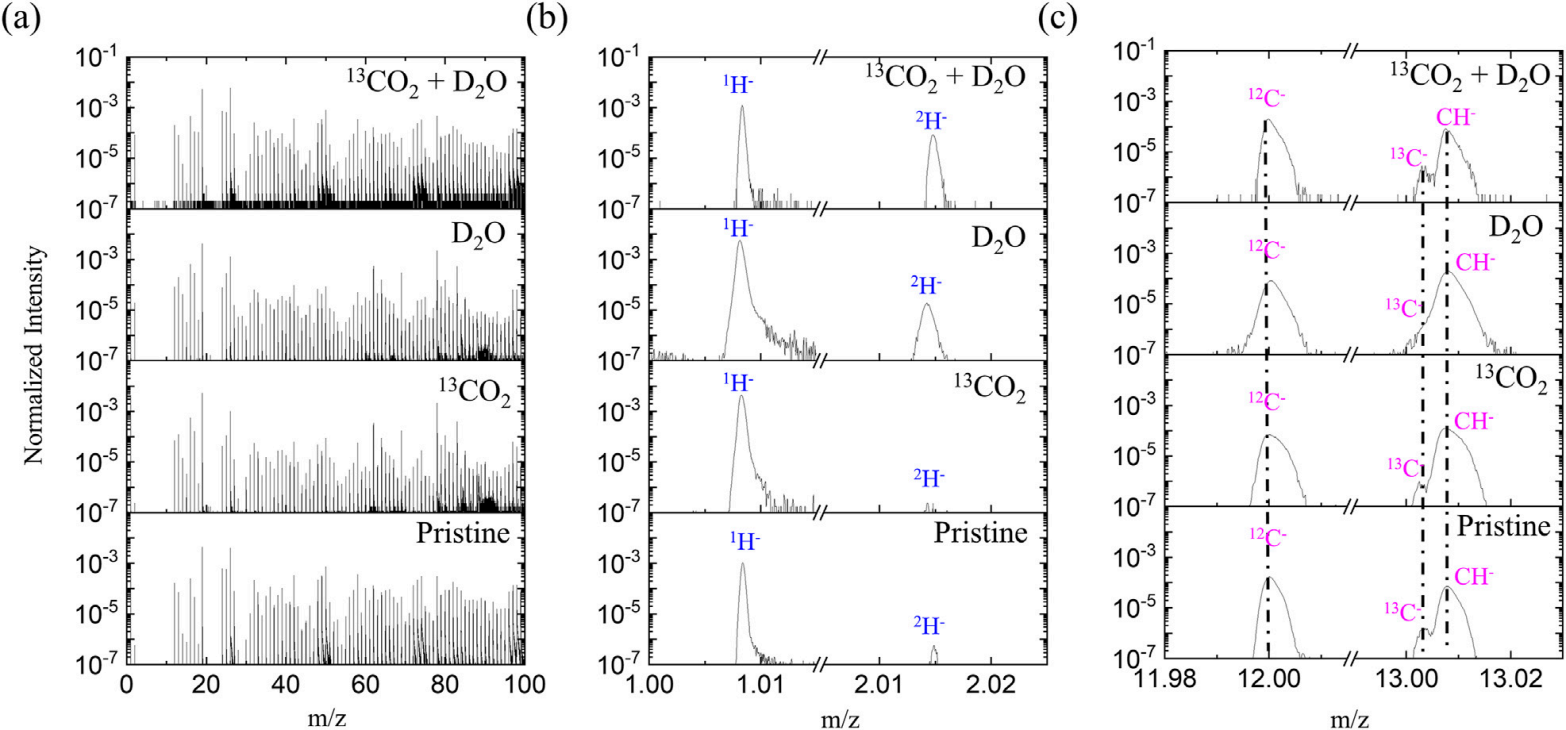
Negative ion ToF-SIMS spectra of a pristine PEEK-ionene sample, after ^13^CO_2_ loading, after D_2_O loading and after ^13^CO_2_ + D_2_O loading, with **(a)** m/z 0–100, **(b)** showing H^-^ and ^2^H^-^, and **(c)** showing ^12^C^-^, ^13^C, and ^12^CH^-^.

Since C^−^ signal is much more sensitive than C^+^ in SIMS analysis, the negative ion mode was prioritized for analysis and comparison. Figure 2 shows the negative ion spectra of a pristine PEEK-ionene membrane, as well as the spectra obtained after loading with ^13^CO_2_, D_2_O separately, and a combination of ^13^CO_2_ + D_2_O. Interestingly, no ^13^C enrichment was observed after loading with ^13^CO_2_ or ^13^CO_2_ + D_2_O. In fact, no significant difference was observed between the whole spectra of the pristine and the ^13^CO_2_ -loaded membranes in a m/z range from 1–800 (Supplementary Figure S2). We repeated measurement multiple times, and the results are consistent: no enrichment of ^13^C was observed. These results suggest that very little ^13^CO_2_ remains in the sample during the cryo ToF-SIMS analysis. In contrast, a clear increase in D^−^ and OD^−^ was observed in the spectra of the D_2_O-loaded and ^13^CO_2_ + D_2_O-loaded samples (Figure 2), suggesting that D_2_O remains in the sample during the cryo ToF-SIMS analysis. These results indicate that the CO_2_-PEEK-ionene interactions are relatively weak, at least weaker than H_2_O-H_2_O interaction in ice (18–20 kJ/mol (Wendler et al., 2010)). This is supported by previous reports showing that ice can be kept under ToF-SIMS analysis at around −110°C (Guo et al., 2020; Cannon et al., 2000), and D_2_O-related signals can be clearly seen during our testing. The choice to use D_2_O instead of H_2_O was primarily to eliminate potential interference from water condensation during the rapid freezing process. Since H_2_O is ubiquitous in the environment, using it could introduce uncertainty in our analysis due to unintended condensation. Additionally, the PEEK-ionene membrane itself contains hydrogen, which would further complicate species differentiation. Using D_2_O allows for clear identification of water-related signals without interference, ensuring more accurate assessment of water-membrane interactions.

It should be noted that NMR results indeed show that ^13^CO_2_ can go into the PEEK-ionene membrane, and the evidence is ^13^C NMR frequency shift if compared to pure ^13^CO_2_ gas (Walter et al., 2023; Chen et al., 2025). However, the ^13^C NMR frequency shift of ^13^CO_2_ molecules is small, only about 0.5–4 ppm, indicating some weak interactions between CO_2_ and PEEK-ionene, qualitatively consistent with our cryo ToF-SIMS result. It is important to mention that a chemical interaction can lead to a large ^13^C NMR frequency shift. For example, N-(2-ethoxyethyl)-3-morpholinopropan-1-amine (EEMPA) can react with CO_2_ to form chemically bound carbamate, where about 35 ppm ^13^C NMR frequency shift can be observed (from about 125 ppm (^13^CO_2_ gas) to about 160 ppm (carbamate)) (Walter et al., 2023; Chen et al., 2025).

**FIGURE 3:**
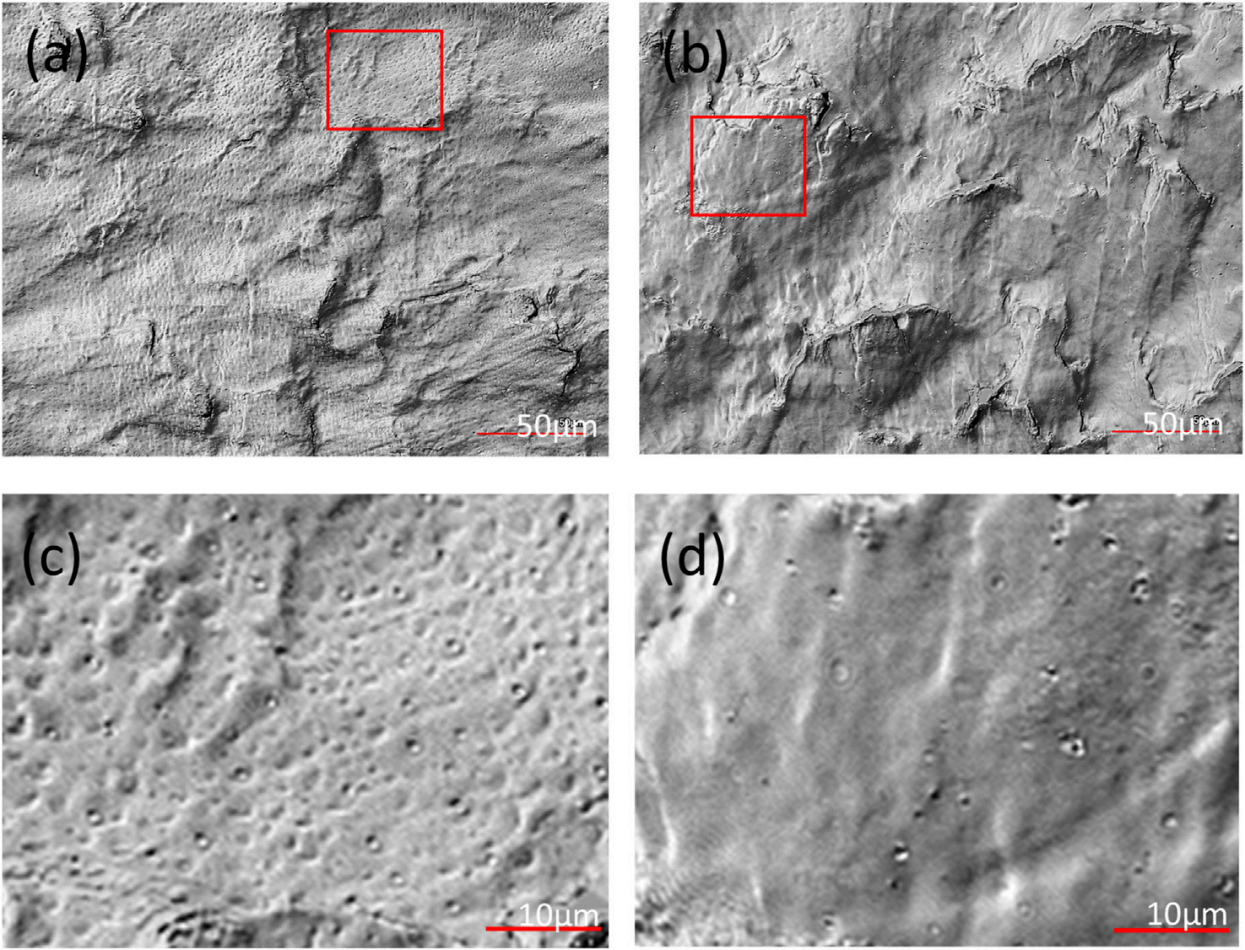
Optical images of **(a, c)** the ^13^CO_2_-loaded PEEK-ionene membrane and **(b, d)** the pristine membrane at different magnifications, with panels **(c, d)** showing zoomed-in views of the regions highlighted by the red boxes in **(a, b)**, respectively, showing the porous structure observed in the PEEK- ionene membrane after CO_2_, loading.

Based on the above results, it is evident that CO_2_ molecules do not engage in any chemical interactions with the PEEK-ionene membrane, only exhibiting van der Waals forces between them. These interactions appear to induce a structural response in the membrane, as the initially flat PEEK-ionene membrane became notably brittle after CO_2_ loading. Figure 3 shows the representative surface optical images of PEEK-ionene membranes with and without ^13^CO_2_ loading, obtained using a Keyence 3D Surface Profiler. Figures 3a, c show the surface of the ^13^CO_2_-loaded membrane, characterized by more pronounced porous features, likely resulting from CO_2_ diffusion into the membrane. Figures 3b, d depict the pristine membrane, displaying a relatively smoother structure with fewer porous characteristics. The change in membrane morphology is expected, as some level of affinity between CO_2_ and the PEEK-ionene membrane is essential to facilitate CO_2_ diffusion into the membrane. Additionally, these interactions must remain weak, as stronger interactions would reduce the mobility of CO_2_ molecules, hindering their diffusion and passage through the PEEK-ionene membrane.

The increase of the D^−^ signal is expected in the D_2_O-loaded membrane, as D_2_O has similar properties with H_2_O, which can be immobilized as ice under high vacuum at −110°C. Since the sample temperature during our cryo ToF-SIMS analysis was approximately −140°C, the D_2_O remained in its ice form throughout the entire analysis. A comparison of the spectra of the pristine PEEK-ionene sample and the D_2_O-loaded sample shows that few new signals were observed except for D^−^, OD^−^, and (D_2_O)_n_OD^−^ signals, indicating that water molecules did not react with any components in the PEEK-ionene membrane or IL, in alignment with our expectation (Supplementary Figure S3).

It should be noted that the D_2_O-related signals, such as D^−^ and OD^−^, are relatively weak in the D_2_O-loaded sample, while they are much stronger in the ^13^CO_2_ + D_2_O-loaded sample. A possible explanation is that the presence of CO_2_ may promote the diffusion of D_2_O into the PEEK-ionene membrane, as CO_2_ can react with H_2_O to form H_2_CO_3_. This explanation is supported by an NMR observation that ^13^CO_2_-D_2_O interaction can be seen in PEEK-ionene membrane (Chen et al., 2025). However, the existence of D_2_O does not appear to facilitate the trapping of ^13^CO_2_ in the PEEK-ionene membrane, as no ^13^C enrichment was observed in the ^13^CO_2_ + D_2_O loaded membrane sample. This is understandable since H_2_CO_3_ is an unstable compound and readily dissociates to release CO_2_. These results suggest that the CO_2_-H_2_O interactions are weaker than the H_2_O-H_2_O interactions. Therefore, it is reasonable to expect that the presence of water vapor would have a limited effect on CO_2_ diffusion in the PEEK-ionene membrane, even with IL loading.

**FIGURE 4:**
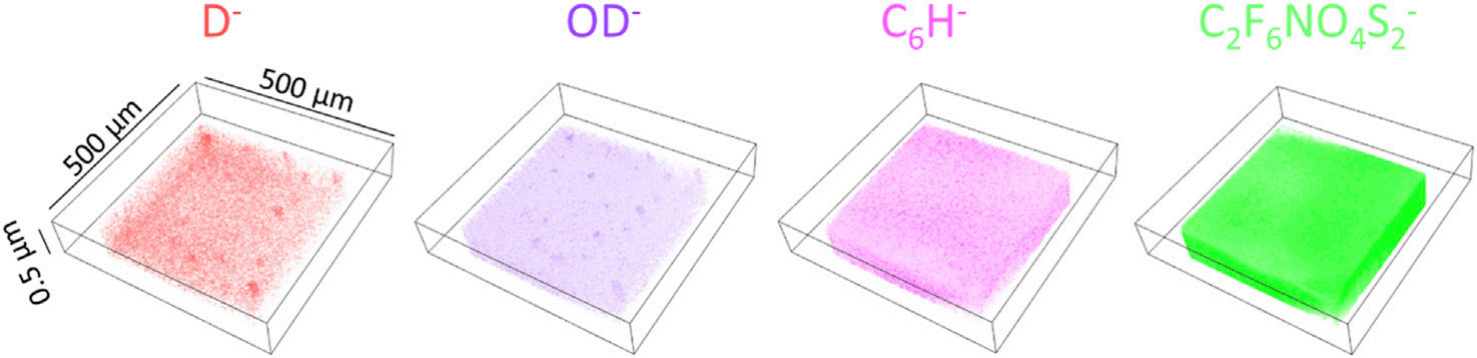
3D images of the characteristic secondary ions on a ^13^CO_2_ + D_2_O loaded PEEK-ionene membrane.

Figure 4 shows 3D images of the D_2_O related peaks (D^−^, OD^−^) and the peaks from PEEK-ionene (C_6_H^−^ and m/z C_2_F_6_NO_4_S_2_^−^), obtained from the D_2_O+^13^CO_2_ loaded membrane. More molecular fragments and peak assignments of this type of PEEK-ionene have been published previously using static ToF-SIMS (Strange et al., 2024b; Strange et al., 2024a). The 3D images of D^−^ and OD^−^ show that D_2_O distribution is relatively even, indicating that water molecules can diffuse into the PEEK-ionene membrane. In addition, there are some D^−^ enrichment locations, suggesting localized water accumulation, possibly in regions such as pores observed in Figure 3. This observation is interesting, because our previous NMR and computer simulation results do suggest some structure changes after CO_2_ loading (Walter et al., 2023; Chen et al., 2025). The 3D images show a stable distribution of D_2_O in the membrane up to a depth of 0.5 µm. Practically, ToF-SIMS molecular imaging analysis of an organic sample can provide a lateral resolution of about 0.2–0.3 µm and a depth resolution better than 5 nm if the imaging analysis mode is applied. In this study, we optimized for high mass resolution rather than imaging mode. Although the spatial resolutions cannot compete with TEM and SEM, ToF-SIMS imaging can provide a unique field-of-view to examine uniformity of the PEEK-ionene membrane. 3D images of the peaks D^−^, OD^−^, C_6_H^−^ and C_2_F_6_NO_4_S_2_^−^ observed from the pristine PEEK-ionene membrane and D_2_O loaded membrane are presented in Supplementary Figure S4 for comparison.

**FIGURE 5:**
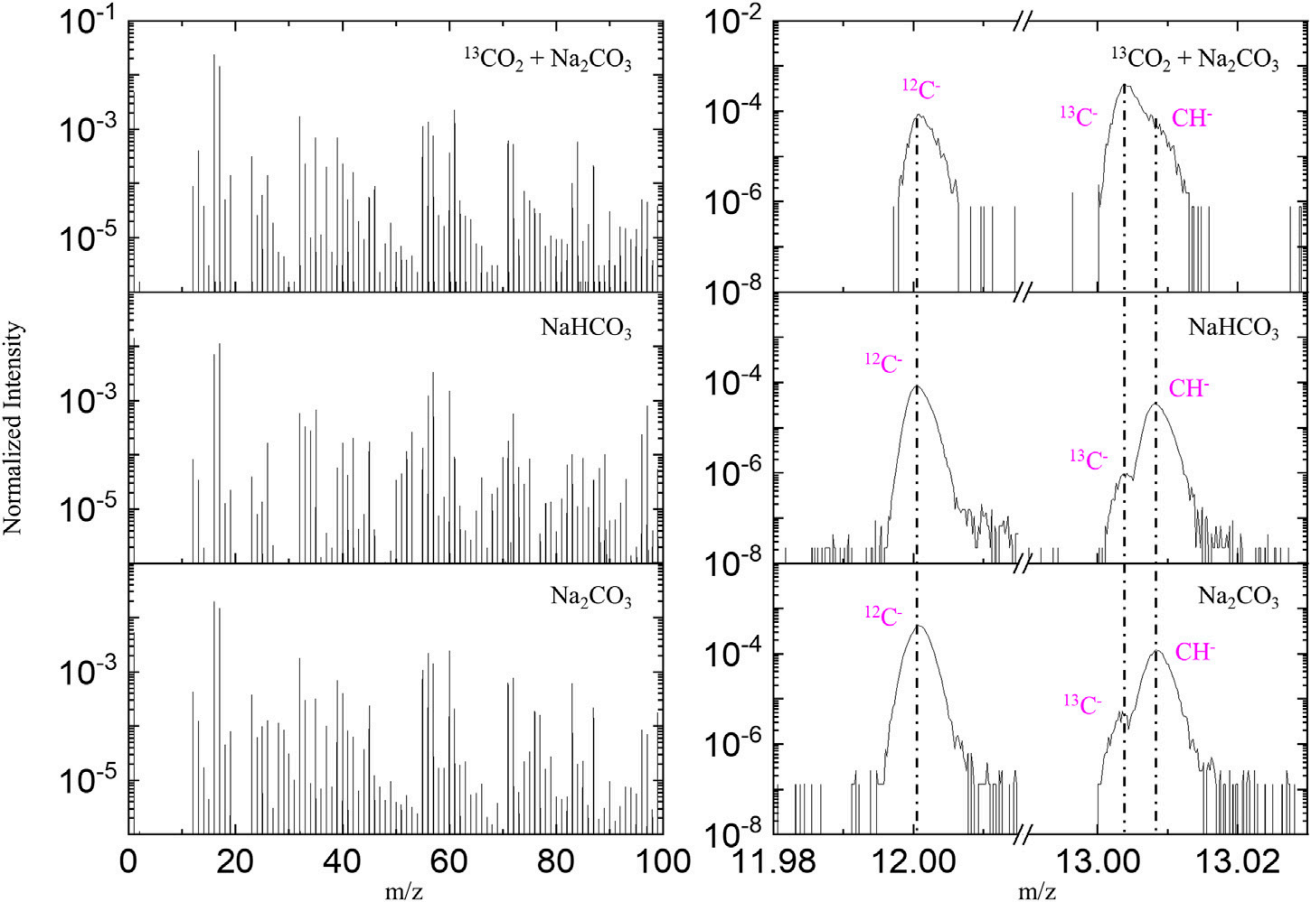
Negative ion TOF-SIMS spectra of a 0.1 M Na_2_CO_3_, a ^13^CO_2_ loaded 0.1 M Na_2_CO_3_, and a 0.1 M NaHCO_3_ aqueous solution, with left panel showing the spectral comparison of m/z 1–100, and right panel highlighting ^12^C^-^, ^13^C^-^, and ^12^CH^-^.

To test if chemically bonded ^13^CO_2_ molecules can be observed during cryo ToF-SIMS testing, we introduced ^13^CO_2_ into a 0.1 M Na_2_CO_3_ aqueous solution for cryo ToF-SIMS analysis. A pristine 0.1 M Na_2_CO_3_ and a 0.1 M NaHCO_3_ aqueous solution samples (both at natural isotopic abundance) were also tested as references. Figure 5 shows the negative ion spectra of these three samples. A significant increase of ^13^C/^12^C ratio was observed in the spectrum of the ^13^CO_2_-loaded Na_2_CO_3_ sample, indicating a chemical reaction effectively captures CO_2_ in an aqueous system. The chemical reaction formula is represented in Equation 1.

$${\text{CO}}_{2}+{\text{Na}}_{2}{\text{CO}}_{3}+H_{2}O\;⇌\;2{\text{NaHCO}}_{3}$$ (1)

The NMR frequency shift of ^13^C in HCO_3_^−^ is about 160–161 ppm (about 169–170 ppm in CO_3_^2−^) (Pironti et al., 2017; Moore et al., 2015), much larger than the NMR frequency shift of ^13^C in ^13^CO_2_ gas (about 125–128 ppm) (Sin et al., 2019; Walter et al., 2023; Chen et al., 2025), indicating that a chemical interaction (normally, hundreds of kJ/mol) results in significant NMR frequency shift (30–40 ppm) compared to van der Waals interactions (normally a few to tens of kJ/mol).

ToF-SIMS analysis offers two distinct advantages: molecular recognition and isotopic identification capabilities. Negative ion ToF-SIMS spectra from a 0.1 M NaHCO_3_, a 0.1 M Na_2_CO_3_ and a ^13^CO_2_-loaded 0.1 M Na_2_CO_3_ aqueous solutions are shown in Supplementary Figure S5. Water clusters (H_2_O)_n_OH^−^ (such as m/z 35, 53, 71, 89, 107) and Na_n_OH_n+1_^–^clusters (such as m/z 57, 97) are clearly observed, indicating that an ice matrix was analyzed. Supplementary Figure S5 shows that after ^13^CO_2_ loading, a number of ^12^CO_x_^−^ related signals decreased while corresponding ^13^CO_x_ signals increased. For example, CO_3_^−^ (m/z 60) signal is strong and HCO_3_^−^ (m/z 61) is weak in the spectra of the NaHCO_3_ and Na_2_CO_3_ samples. As a comparison, CO_3_^−^ (m/z 60) signal is weak but ^13^CO_3_^−^ (m/z 61) is strong in the spectrum of ^13^CO_2_-loaded Na_2_CO_3_ sample. Similarly, Na_2_CO_3_OH^−^ (m/z 123), Na_3_(CO_3_)_2_^−^ (m/z 189) and (Na_2_CO_3_)_2_OH^−^ (m/z 229) are strong in the spectra of the NaHCO_3_ and Na_2_CO_3_ samples, while the intensities of Na_2_^13^CO_3_OH^−^ (m/z 124), Na_3_(^13^CO_3_)_2_^−^ (m/z 191), and (Na_2_^13^CO_3_)_2_OH^−^ (m/z 231) are prominent in the spectrum of ^13^CO_2_-loaded Na_2_CO_3_ sample.

This isotopic analysis reveals that the ^13^C/^12^C ratio in the Na_2_CO_3_ solution increases from a natural abundance of approximately 1.1% to 267.2% after loading with ^13^CO_2_ (Table 1). Although a 1:1 ^13^C/^12^C ratio might be expected from the reaction Equation 1, the data show a more complete exchange of ^12^C by ^13^C. This observation can be explained by the reversible nature of the reaction, allowing the excess ^13^CO_2_ to continuously replace the original CO_3_^2−^ in the solution until the ^13^C/^12^C ratio equilibrates between the gas and liquid phases. Based on this ^13^C enrichment, we estimate that about 44.9% of the carbon atoms in the original Na_2_CO_3_ solution were replaced by ^13^CO_2_.

**TABLE 1:** Isotopic ratios of^13^C^−^/^12^C^−^ and D^−^/H^−^.

Sample	^13^C^−^/^12^C^−^ (%)^a^	D^−^/H^−^ (%)^a^
Pristine PEEK-ionene	1.2 ± 0.2	0.012 ± 0.004
PEEK-ionene +^13^CO_2_	1.2 ± 0.2	0.010 ± 0.005
PEEK-ionene + D_2_O	1.2 ± 0.2	0.33 ± 0.08
PEEK-ionene + D_2_O+^13^CO_2_	1.2 ± 0.2	9.7 ± 0.2
Na_2_CO_3_ +^13^CO_2_	267.2 ± 16.7	0.02 ± 0.01
NaHCO_3_	1.1 ± 0.1	0.015 ± 0.005
Na_2_CO_3_	1.01 ± 0.08	0.011 ± 0.003

^a^ Rounding to the significant figures of the uncertainty.

**FIGURE 6:**
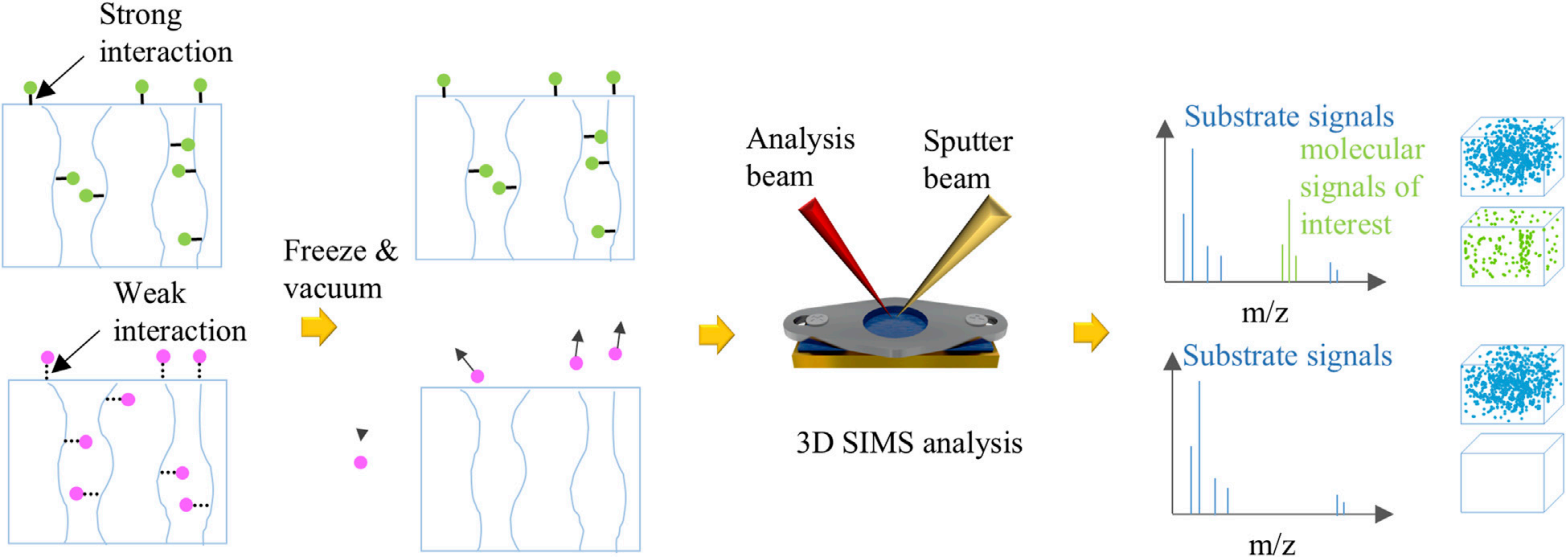
A schematic illustration of investigation of various interactions between gas molecules and samples using cryo ToF-SIMS. When gas molecules can form strong interaction with samples, e.g., equal to or stronger than hydrogen bond in ice, the gas molecules can stay in the sample during cryo ToF-SIMS testing. Two unique advantages are TOF-SIMS spectra can provide molecular information to distinguish the product, and 3D imaging can track gas molecules distribution in the sample. Also, combined with isotopic labeling, it is possible to quantify trace gas molecules in the samples. If only weak interactions exist between gas molecules and the samples, such as van der Waals forces, the gas molecules escape into vacuum during cryo TOF-SIMS testing, and little change can be observed before and after gas loading.

Figure 6 presents a schematic representation of the interaction mechanisms between CO_2_ and IL-loaded PEEK-ionene membranes, as evaluated using cryo ToF-SIMS. The figure highlights the distinction between strong and weak interactions. When strong interactions occur, such as chemical bonding, cryo ToF-SIMS can detect the relevant molecular signals, confirming the presence of chemical bonds. On the other hand, if only weak interactions, such as London dispersion forces, are present, cryo ToF-SIMS analysis confirms the absence of chemical bonding, as no significant molecular signals corresponding to strong interactions are observed. This method proves effective in determining whether CO_2_ is chemically bound to the membrane or merely interacting through weak van der Waals forces. This method can be broadly applied to assess various gas-material interactions. For instance, the hydrogen bond between water molecules (∼18–20 kJ/mol) serves as a reference point for evaluating interaction strengths. If an interaction is weaker than a hydrogen bond, small molecules can sublimate under high vacuum, even at low temperatures (−110 to −140°C). Stronger interactions, such as chemical bonds, can retain gas molecules in the matrix. The interaction strength can be further assessed by systematically increasing the temperature, providing a powerful tool to quantify gas-material interactions in various CO_2_ capture systems, including ionic liquid-loaded membranes like PEEK-ionene. This method serves as a screening tool to qualitatively evaluate gas-material interactions and determine whether chemical bonding or a reaction has occurred. For a more definitive assessment, techniques such as Infrared (IR) spectroscopy or calorimetry can be used. If multiple gases interact with the material and are expected to exhibit interactions weaker than hydrogen bonds, lowering the temperature may help distinguish their interactions with the membrane. A relevant cryogenic technique using liquid He is discussed in other group’s work (Suzuki and Iimura, 2024).

The results in this work hold significant potential for post-combustion CO_2_ capture research. For example, the interaction of SO_x_ (sulfur oxides) and NO_x_ (nitrogen oxides) with CO_2_ capture solvents like EEMPA (Zheng et al., 2020) can form heat-stable salts with SO_x_ and NO_x_. This methodology can help further the understanding of SO_x_ and NO_x_ sorption in carbon capture media (Wang et al., 2021; Boumghar et al., 2020) as detecting and quantifying low concentrations of SO_x_ and NO_x_ is challenging for conventional experimental methodologies like NMR and IR. Given the excellent detection limits of ToF-SIMS (ppm to ppb level) (Lockyer et al., 2024; Benninghoven, 1994), we expect that combining isotopic labeling (e.g., ^15^NOx, S^18^O_2_) with cryo-ToF-SIMS will enable confident quantification of these gases in CO_2_ capture systems, providing critical insights into mechanisms of heat stable salt formation. Future studies will focus on this approach, offering a pathway to better understand the role of these flue gas components in solvent-based carbon capture.

Moreover, controlling the interaction strength between CO_2_ and PEEK-ionene membranes is crucial for optimizing CO_2_ permeability and diffusion. Since CO_2_ can react with H_2_O to form carbonic acid (H_2_CO_3_), the introduction of basic functional groups like -NH_2_ into the PEEK-ionene membrane could significantly influence CO_2_-membrane interactions. Basic groups can promote chemical reactions with CO_2_, leading to stronger and less reversible interactions. This would trap CO_2_ within the membrane, reducing its permeability and overall efficiency of gas separation. Therefore, our experimental observations suggest that avoiding basic conditions is essential to maintain optimal membrane performance.

## 4 Conclusion

In this study, ^13^CO_2_ and D_2_O was introduced to the IL-loaded PEEK-ionene membranes and cryo ToF-SIMS was utilized to examine interactions among CO_2_, water and the membrane. A special sample block was designed and fabricated that allows direct sample cooling under ^13^CO_2_ loading condition and facilitates subsequent sample transferring under low temperature with little ice condensation on the sample surface. While previous NMR data show that ^13^CO_2_ can be absorbed into the PEEK-ionene membrane under ambient condition, little enhancement of ^13^C/^12^C ratio (compared to natural abundance) was observed in our cryo ToF-SIMS data, suggesting that the CO_2_-membrane interactions are weak, leading to CO_2_ vaporization even at low temperatures (−140°C) under vacuum condition. In contrast, cryo ToF-SIMS detected D_2_O in the heavy water-loaded membrane, showing a relatively homogeneous 3D distribution within the membrane with some highlighted locations, possibly in the pores induced by CO_2_ loading. This implies that CO_2_-membrane interaction is weaker than the water-membrane interaction, as well as hydrogen bond in water (approximately 18–20 kJ/mol), which is consistent with our prior NMR and computer simulation results. Additionally, cryo ToF-SIMS did not detect ^13^CO_2_ in the membrane even with the co-existence of D_2_O, indicating weak CO_2_-D_2_O interactions, thereby suggesting minimal interference from water vapor on CO_2_ diffusion. As a comparison, our ToF-SIMS data show that ^13^CO_2_ can readily react with a basic Na_2_CO_3_ aqueous solution to form NaH^13^CO_3_, indicating a chemical interaction can effectively retain CO_2_ gas molecules under cryogenic conditions. This observation suggests the importance of controlling the conditions for CO_2_-membrane interactions. Specifically, it is recommended to avoid basic conditions when using the PEEK-ionene membrane for CO_2_ separation, as such conditions may lead to unwanted interactions, potentially compromising the membrane’s performance and the efficiency of CO_2_ separation. These observations suggest that our cryo ToF-SIMS method is a unique tool to examine interaction strength between various gas-material interactions. Also, the molecular recognition capability of ToF-SIMS can effectively elucidate the molecular structures of new chemical species after gas loading. Furthermore, a combination of isotopic labeling and cryo ToF-SIMS can be used to quantify impurities or poisonous gases in water-lean CO_2_ capture solvents. These advantages position cryo ToF-SIMS as a powerful analytical tool for advancing CO_2_ capture research and industrial application.

## Data Availability

The raw data supporting the conclusions of this article will be made available by the authors, without undue reservation.
