# Supplementary material for: Investigating intermolecular interactions among CO2, water and PEEK-ionene membrane using cryo ToF-SIMS and isotopic labeling
Source: Front Chem. 2025 Mar 14;13:1564084. doi: 10.3389/fchem.2025.1564084 (PMC11949930; doi:10.3389/fchem.2025.1564084)
Supplement: Supplementary file 1 [file DataSheet1.docx]

Supplementary Material

Investigating intermolecular interactions among CO_2_, water and PEEK-ionene membrane using cryo ToF-SIMS and isotopic labeling

**Jennifer Yao^1^*, Jeffrey A. Dhas^1^, Lyndi E. Strange^1^, Jason E. Bara^2^, Sudhir Ravula^2^, Eric D. Walter^1^, Ying Chen^1^, David J. Heldebrant^1,3^, and Zihua Zhu^1*^**

^1^Pacific Northwest National Laboratory, Richland, WA 99352 USA

^2^The University of Alabama: Tuscaloosa, AL, 35487 USA

^3^Washington State University Department of Chemical Engineering, Pullman, WA 99163, USA

*** Correspondence:**Jennifer Yao [jennifer.yao@pnnl.gov](mailto:jennifer.yao@pnnl.gov);

Zihua Zhu [zihua.zhu@pnnl.gov](mailto:zihua.zhu@pnnl.gov)


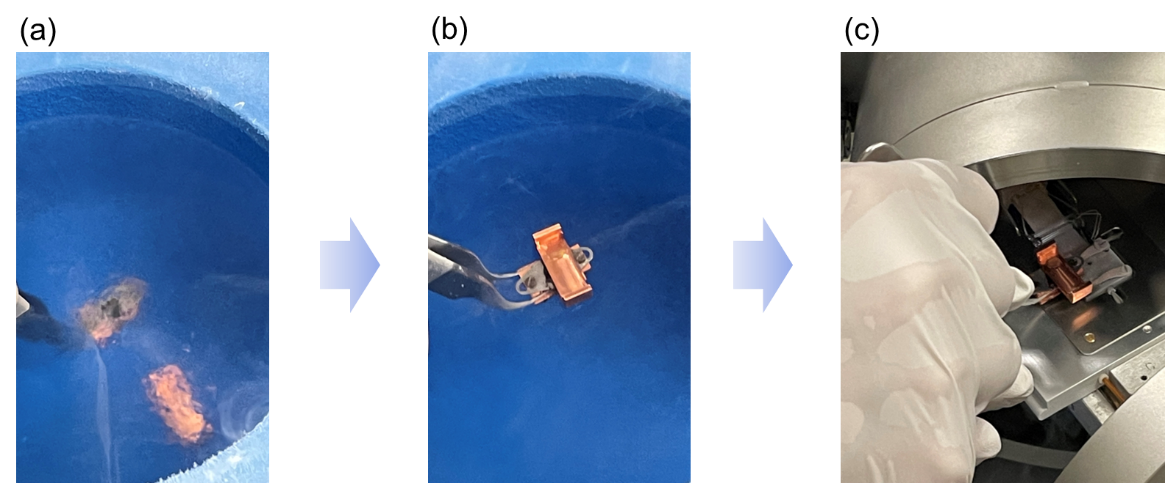


**Figure S1**. Three photos show the process of transferring a cryo-sample assembly from a liquid N_2_ cooling dewar onto a pre-cooled ToF-SIMS heating/cooling sample holder. (a) A sample assembly and a copper cover piece are immersed in liquid N_2_. (b) Add the copper cover piece on top of the analysis area (to avoid any ice condensation during sample transferring), and then take the whole assembly out. (c) In less than 10 seconds, the whole sample assembly can be inserted into a pre-cooled ToF-SIMS heating/cooling sample holder in the load lock, and the load lock door can be closed for pumping.


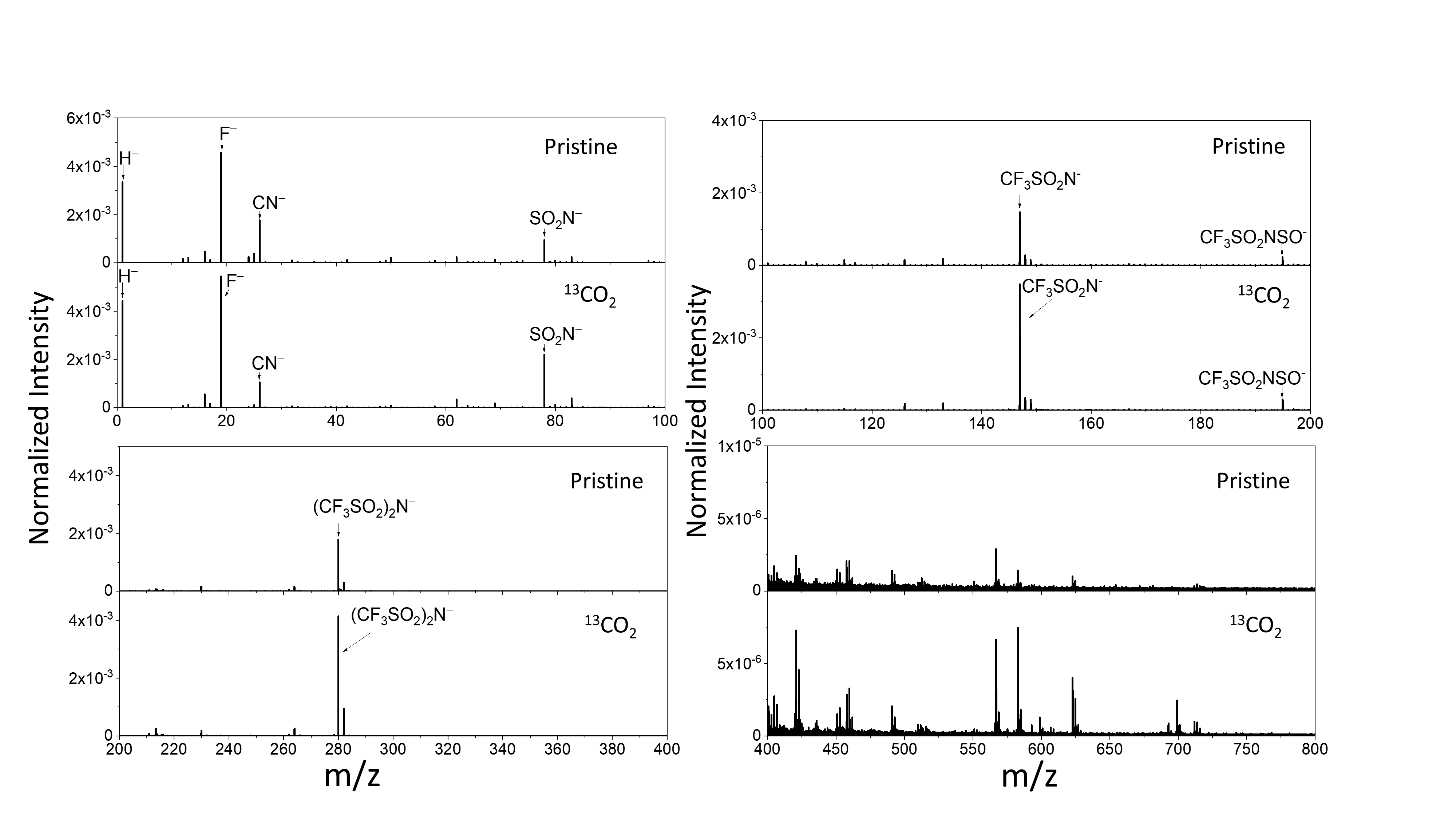
**Figure S2**. A detailed comparison of the negative ion ToF-SIMS spectra from a pristine and a ^13^CO_2_ loaded PEEK-ionene sample.


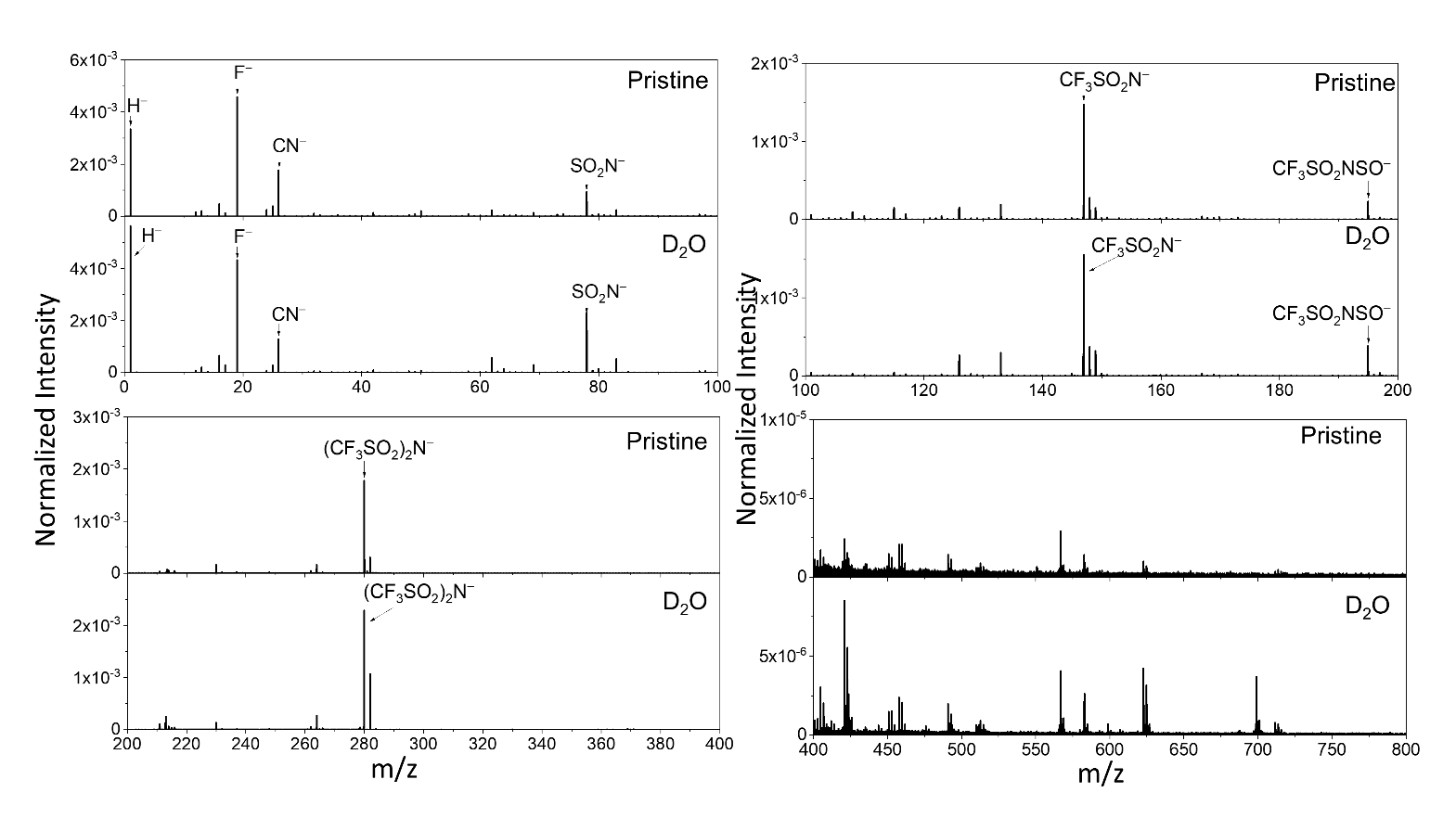


**Figure S3**. A detailed comparison of the negative ion ToF-SIMS spectra from a pristine and a D_2_O loaded PEEK-ionene membranes. m/z 1-100, 100-200, 200-400, and 400-800


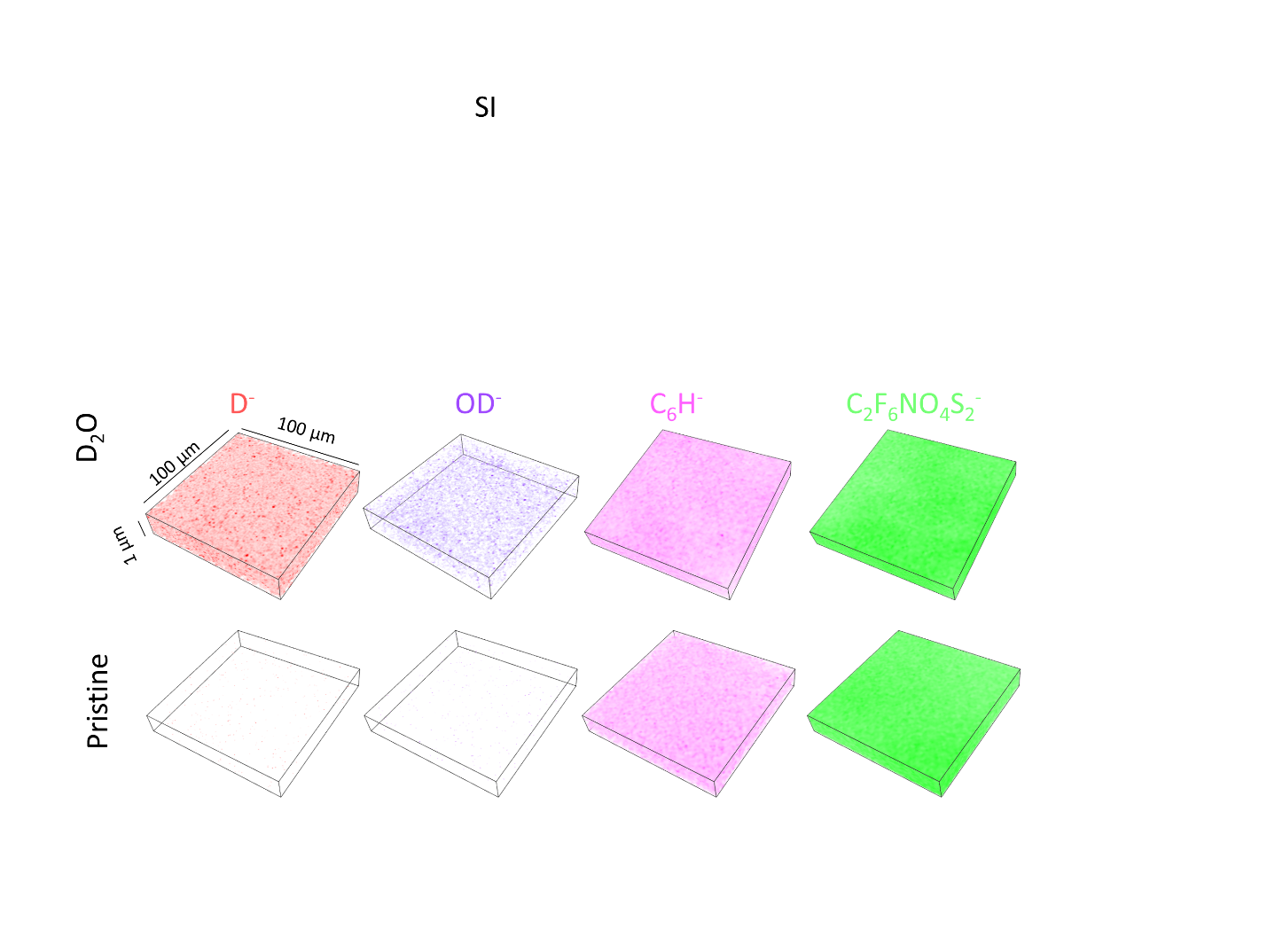


**Figure S4**. 3D images of the characteristic secondary ions in D_2_O loaded and pristine PEEK-ionene membranes


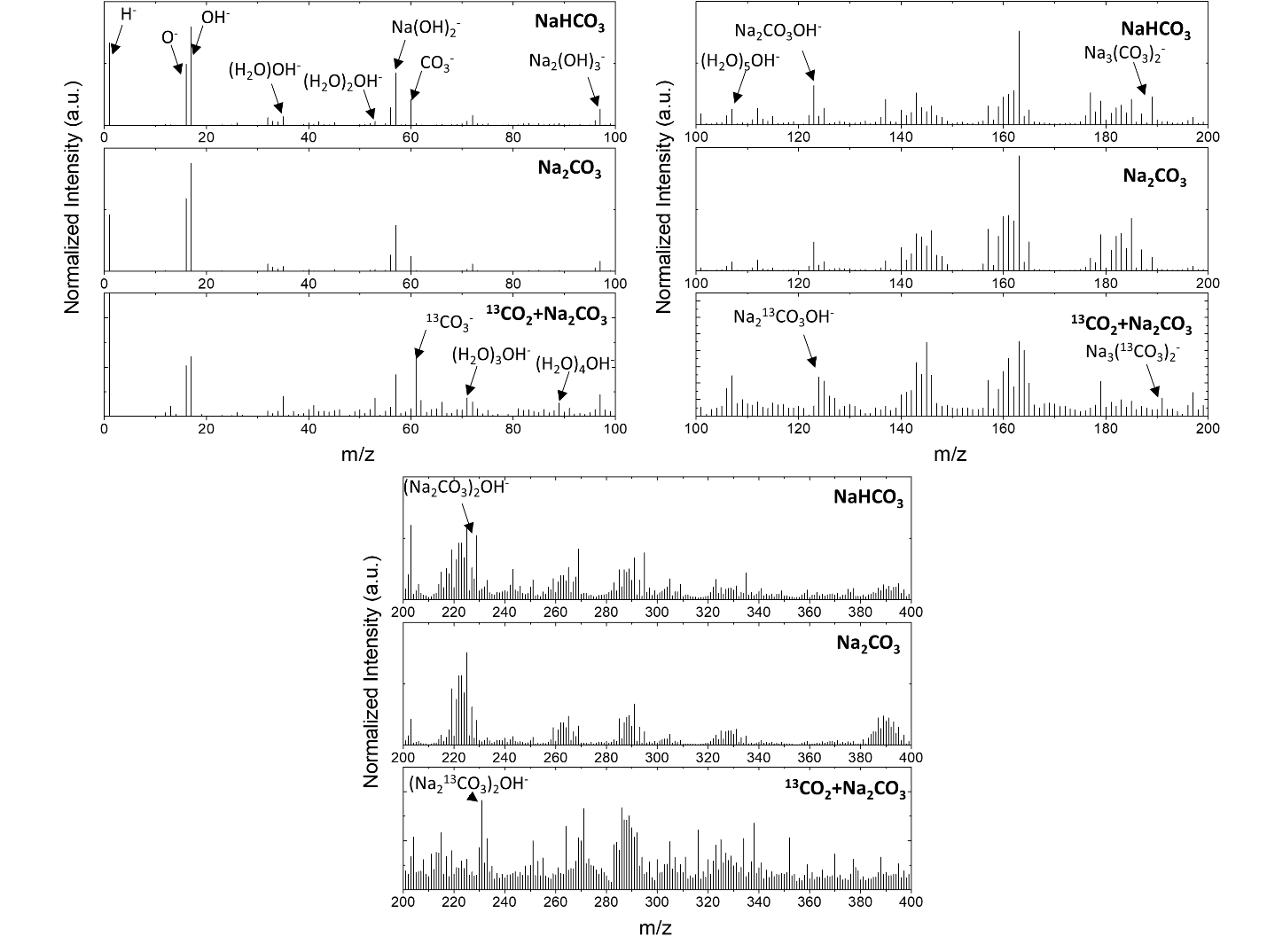


**Figure S5**. A detailed comparison of the negative ion ToF-SIMS spectra from a 0.1 M NaHCO_3_, a 0.1 M Na_2_CO_3_ and a ^13^CO_2_ loaded 0.1 M Na_2_CO_3_ aqueous solutions. A linear signal intensity (y) scale is used here so that CO_x_ and ^13^CO_x_-related signals can be clearly differentiated. Water clusters (H_2_O)_n_OH^-^ (such as m/z 35, 53, 71, 89, 107…) and Na_n_OH_n+1_ ^–^clusters (such as m/z 57, 97…) are clearly observed, indicating that an aqueous environment was analyzed. CO_3_^-^ (m/z 60) signal is strong and HCO_3_^-^ (m/z 61) is weak in the spectra of the NaHCO_3_ and Na_2_CO_3_ samples. As a comparison, CO_3_^-^ (m/z 60) signal is weak but ^13^CO_3_^-^ (m/z 61) is strong in the spectrum of ^13^CO_2_ loaded Na_2_CO_3_ sample. Also, Na_2_CO_3_OH^-^ (m/z 123), Na_3_(CO_3_)_2_^-^ (m/z 189) and (Na_2_CO_3_)_2_OH^-^ (m/z 229) are strong in the spectra of the NaHCO_3_ and Na_2_CO_3_ samples, while Na_2_^13^CO_3_OH^-^ (m/z 124), Na_3_(^13^CO_3_)_2_^-^ (m/z 191), and (Na_2_^13^CO_3_)_2_OH^-^ (m/z 231) are strong in the spectrum of ^13^CO_2_ loaded Na_2_CO_3_ sample.
